# 胸腔积液及沉淀物碳酸酐酶IX检测对肺癌的诊断价值

**DOI:** 10.3779/j.issn.1009-3419.2015.11.06

**Published:** 2015-11-20

**Authors:** 李娜 彭, 小娥 王, 殿胜 钟, 倩 王, 畅 刘

**Affiliations:** 300052 天津，天津医科大学总医院肿瘤科 Department of Medical Oncology, Tianjin Medical University General Hospital, Tianjin 300052, China

**Keywords:** 肺肿瘤, 胸腔积液, 沉淀物, 碳酸酐酶IX, 诊断, Lung neoplasms, Pleural effusion, Pleural effusion sediment, Carbonic anhydrase IX, Diagnosis

## Abstract

**背景与目的:**

碳酸酐酶IX(carbonic anhydrase IX, CAIX)在肺癌等多种恶性肿瘤组织中广泛表达，前期研究证明，肺癌患者血清可溶性CAIX(soluble form of carbonic anhydrase IX, s-CAIX)含量明显高于健康者。本研究检测肺癌恶性胸腔积液中s-CAIX含量和沉淀物中CAIX表达情况，探讨其对肺癌的诊断价值。

**方法:**

运用EILSA检测29例肺癌恶性胸腔积液和27例结核性胸腔积液中s-CAIX含量。运用免疫组化法检测21例肺癌恶性胸腔积液沉淀物和6例良性胸腔积液沉淀物中CAIX表达。绘制胸腔积液s-CAIX诊断肺癌恶性胸腔积液的ROC曲线。

**结果:**

肺癌恶性胸腔积液较结核性胸腔积液中s-CAIX含量明显增高(*P*＜0.05)。胸腔积液s-CAIX诊断肺癌恶性胸腔积液的受试者工作特征(receiver operating characteristic, ROC)曲线下面积为0.761，当胸腔积液中s-CAIX阈值为109.135 pg/mL时，敏感度和特异度分别为92.3%和58.3%。良性胸腔积液沉淀物组中CAIX表达均阴性，肺癌恶性胸腔积液沉淀物中CAIX表达阳性率66.67%。

**结论:**

胸腔积液及其沉淀物CAIX检测有助于肺癌恶性胸腔积液诊断，且具有较高的敏感性和特异性。

## Abstract (en)

**Background and objective:**

Carbonic anhydrase IX (CAIX) is widely expressed in a variety of malignant tumors, including-lung cancer. Our previous study has shown that the serum level of soluble form of carbonic anhydrase IX (s-CAIX) was significantly higher in patients with lung cancer than that in the healthy group. The aim of this study is to detect the s-CAIX level in the pleural effusion and its sediment, and to evaluate the significance of CAIX detection in the diagnosis of lung cancer.

**Methods:**

The s-CAIX level in pleural effusion of 29 lung cancer patients and 27 patients with tuberculosis was detected by ELISA. The expression of CAIX in the pleural effusion sediment of 21 lung cancer patients with malignant pleural effusion and 6 patients with benign pleural effusion was examined by immunohistochemistry. With pathological diagnosis as the gold standard, receiver operating characteristic (ROC) curve of pleural effusion s-CAIX was established for the diagnosis of lung cancer with malignant pleural effusion.

**Results:**

The s-CAIX level in the malignant pleural effusion was significantly higher than that in the tuberculosis group (*P* < 0.05). The AUC of pleural effusion s-CAIX level was 0.761. At a threshold level of 109.135 pg/mL, sensitivity and specificity were 92.3% and 58.3%, respectively. The CAIX expression in all samples of the benign pleural effusion sediment was negative. The positive rate of CAIX expression in malignant pleural effusion sediment was 66.67%.

**Conclusion:**

Detection of CAIX in the pleural effusion and its sediment exhibits high sensitivity and specificity, and is helpful in diagnosis of lung cancer with malignant pleural effusion.

胸腔积液是临床常见病症。在我国，渗出性胸腔积液最常见的病因是结核性胸膜炎和胸膜的原发或转移性肿瘤，其中，最易转移至胸膜的恶性肿瘤是肺癌^[1]^。细胞学检测是鉴别良、恶性胸腔积液常用手段，但其阳性率较低，胸腔积液沉淀物切片诊疗率较细胞学明显增高^[2]^，且可运用免疫组化染色进一步提高诊疗率，便于病因诊断。

碳酸酐酶为一类含锌的金属蛋白酶，其生理作用为可逆的催化：CO_2_+H_2_O→H++HCO_3-_，他们的主要功能是维持机体的酸碱平衡，其次参与其他生理过程，如骨再吸收、体液的产生等^[3]^。碳酸酐酶家族共有15种成员^[4]^，其中碳酸酐酶IX(carbonic anhydrase IX, CAIX)不仅参与上述经典的调节酸碱度的生理过程，还参与肿瘤细胞的增殖、细胞粘附和浸润^[5, 6]^。

CAIX为跨膜蛋白，由细胞外部分N末端信号肽和细胞外区，及细胞内部分跨膜区和细胞内C末端构成^[7]^，其胞外部分可被蛋白酶水解，形成可溶性碳酸酐酶IX(soluble form of carbonic anhydrase IX, s-CAIX)释放到血液中^[8]^。我们的前期试验证明，肺癌患者血清s-CAIX含量明显高于健康对照组^[9]^。本研究旨在探索肺癌胸膜转移的胸腔积液中s-CAIX含量及胸腔积液沉淀物中CAIX表达情况，探索其对良、恶性胸腔积液的诊断价值。

## 1 对象和方法

### 1.1 研究对象

选取2012年6月-2013年3月于我院呼吸科及肿瘤科住院确诊肺癌胸膜转移导致恶性胸腔积液患者作为肺癌胸腔积液组，共29例[男性16例，女性13例，年龄(69±11)岁]。全部经胸腔积液沉淀物病理或液基细胞学检查确诊，包括鳞癌1例、腺癌27例、病理类型不明确1例。对其中21例患者的胸腔积液沉淀物进行了免疫组化检测。胸腔积液收集前，患者未接受放疗、化疗等任何抗肿瘤治疗。选取自2012年6月-2013年3月于我院呼吸科确诊结核性胸膜炎患者27例结核性胸腔积液组作为肺癌胸腔积液组[男性21例，女性6例，年龄(56±21)岁]。对其中6例患者的胸腔积液沉淀物进行了免疫组化检测。

### 1.2 研究方法

标本的采集和处理过程如下：上述患者行常规胸腔置管引流术，接一次性引流袋。抽取胸腔积液标本3 mL-4 mL于红色普通血清管内，于4 ℃条件下3, 000 r/min离心10 min，取上清液置于1.5 mL离心管内，-40℃条件下储存。于检测前常温下一次性解冻所有标本。引流胸腔积液500 mL-1, 000 mL时，将引流袋静置2 h-6 h，用镊子将沉淀物取出，置于10%的中性甲醛溶液中固定，送病理科。主要试剂如下：CAIX定量测定试剂盒购自武汉华美生物工程有限公司。CIAX的单克隆抗体购自香港Abcam公司。检测方法：采用酶联免疫吸附测定(enzyme linked immunosorbent assay, ELISA)法检测患者胸腔积液中s-CAIX含量，具体操作严格按照说明书执行。采用免疫组化法测定胸腔积液沉淀物中CAIX的表达。

### 1.3 统计学方法

应用SPSS 17.0统计软件分析处理，对肺癌胸腔积液组和结核性胸腔积液组数据进行正态性检验，均符合正态分布，采用两独立样本*t*检验，以*P*＜0.05表示差异具有统计学意义。绘制胸腔积液s-CAIX诊断肺癌恶性胸腔积液的受试者工作特征(receiver operating characteristic, ROC)曲线并计算曲线下面积及各坐标的敏感度和特异度。

## 2 结果

### 2.1 肺癌胸腔积液组和结核性胸腔积液组胸腔积液中s-CAIX含量

两组患者性别无统计学差异(*P*＞0.05)，年龄有差异(*P*＜0.05)。肺癌组胸腔积液中s-CAIX含量为(241.61±136.74)pg/mL，结核组胸腔积液中s-CAIX含量为(134.61±108.07)pg/mL，肺癌胸腔积液中CAIX含量明显高于结核组，两组间差异有统计学意义(*t*=3.052, *P*=0.040)(图 1)。

**1:**
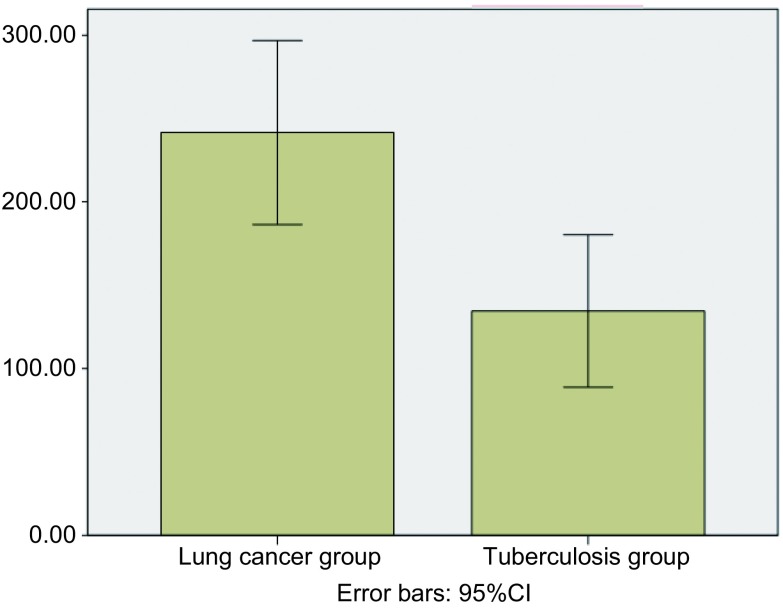
肺癌胸腔积液组与结核性胸腔积液组胸腔积液中s-CAIX含量比较(pg/mL) Comparison of s-CAIX level in the pleural effusion between lung cancer group and tuberculosis group (pg/mL); s-CAIX: soluble form of carbonic anhydrase IX.

### 2.2 绘制胸腔积液s-CAIX诊断肺癌恶性胸腔积液的ROC曲线

以病理诊断为金标准，绘制胸腔积液s-CAIX诊断肺癌恶性胸腔积液的ROC曲线，计算曲线下面积及各阈值的敏感度和特异度。胸腔积液s-CAIX曲线下面积为0.761(*P*=0.002)，面积的95%CI：0.622-0.901。当胸腔积液中s-CAIX阈值为109.135 pg/mL时，敏感度和特异度分别为92.3%、58.3%(图 2)。

**2:**
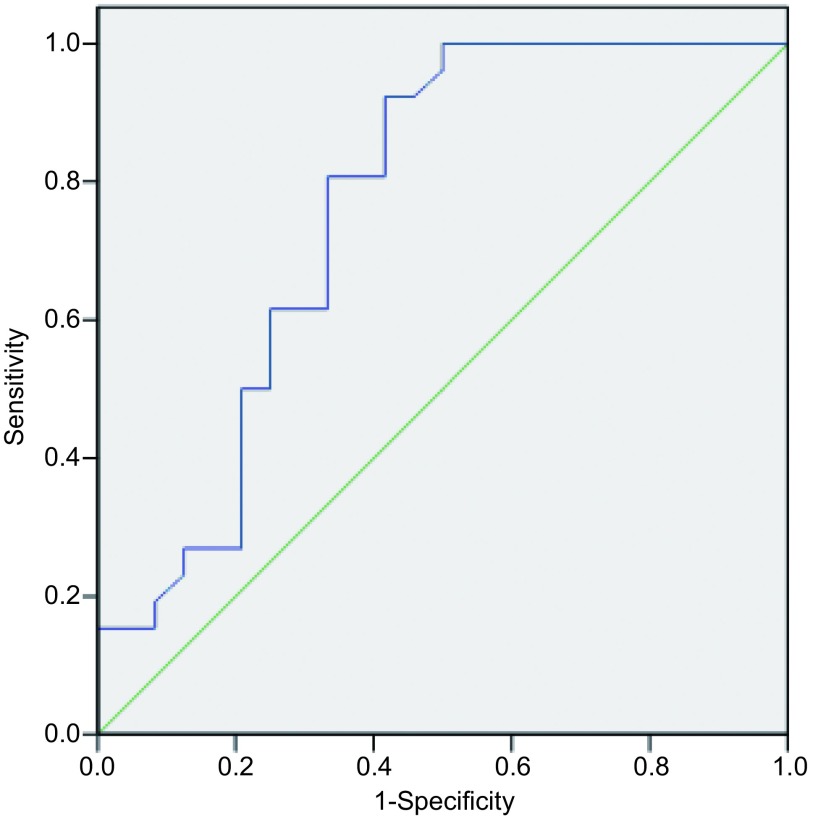
胸腔积液s-CAIX诊断肺癌恶性胸腔积液的ROC曲线 The ROC curve of s-CAIX level in the pleural effusion in the diagnosis of lung cancer with malignant pleural effusion. ROC: receiver operating characteristic.

### 2.3 胸腔积液沉淀物中CAIX表达

对肺癌组与结核组胸腔积液沉淀物标本进行免疫组化染色，探索CAIX在两组标本中的表达情况。因CAIX为跨膜蛋白，则以细胞膜着色判定为阳性。结果显示肺癌组共14例(66.67%)标本可见CAIX表达，但结核组中未见有着色细胞，即CAIX表达阳性率为0(图 3)。

**3:**
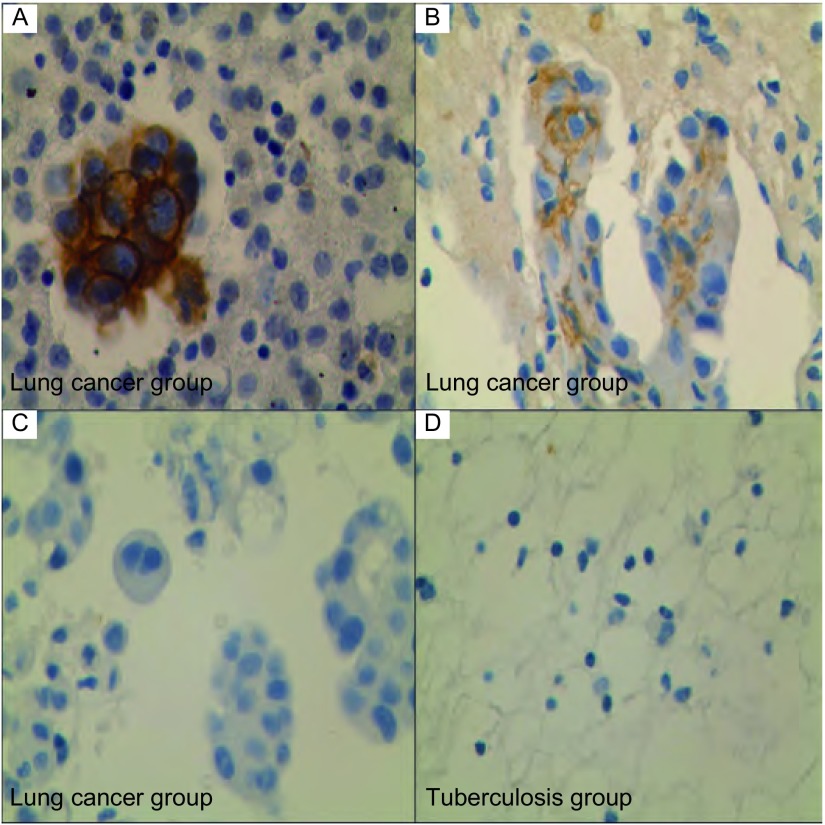
400倍显微镜下胸腔积液沉淀物CAIX免疫组化染色图片。A和B示肺癌组胸腔积液沉淀物免疫组化染色后细胞膜着色的标本；C示肺癌组免疫组化染色后细胞膜未着色标本；D示结核组胸腔积液沉淀物免疫组化染色后细胞膜未着色标本。 Immuno-staining of CAIX expression in the cells of the pleural effusion sediment by immunohistochemistry (×400). Fig A and B demonstrate that the cytomembrane of pleural effusion sediment was dyed by immune-staining of CAIX in the lung cancer group. Fig C show that the cytomembrane was not dyed by immunohistochemistry in the lung cancer group. Fig D reveals that the cytomembrane was not dyed by immunohistochemistry in the tuberculosis group.

## 3 讨论

临床工作中常用的胸腔积液常规、生化检测，对于良、恶性的鉴别诊断有一定局限性。胸腔积液细胞学检查是诊断恶性胸腔积液最简便的病理学依据，但其敏感性仅为50%-60%，假阴性率为31.5%^[10]^。当胸腔积液中肿瘤细胞少、肿瘤细胞失去正常形态，与反应性间皮细胞难以区别时，细胞学检查的缺陷暴露无疑。胸腔镜及开胸肺活检可大大提高恶性胸腔积液的诊断率，达92%以上^[11]^，但上述检查为有创性检查，患者痛苦大且并发症多，部分医疗机构难以实施，在临床工作中不能广泛推广。肿瘤标记物，如CA125^[12]^、CEA^[13]^等，它们在结核性胸腔积液中也可增高。

CAIX属于碳酸酐酶家族中一员，是一种新型的肿瘤抗原。他位于抑癌基因VHL(von Hippel-Lindau)下游，由缺氧诱导因子(hypoxia-inducible factor, HIF)调控表达。在低氧、抑癌基因VLH(von Hippel-Lindau)突变或甲基化、表皮生长因子受体(epidermal growth factor receptor, EGFR)活化等情况下，CAIX表达增加^[14-17]^。

CAIX参与细胞的代谢过程，调节细胞内外酸碱度，并且在肺癌、肾癌、宫颈癌、外阴癌、乳腺癌等多种恶性肿瘤组织中广泛表达，却仅在少量正常组织中低表达^[18-22]^。因此，CAIX对良恶性疾病的鉴别，特异性较高。目前，CAIX已被认为是肾透明细胞癌的诊断标志物^[23-25]^。

本研究运用ELISA检测胸腔积液中s-CAIX含量，发现肺癌胸膜转移患者胸腔积液中s-CAIX含量明显高于结核性胸腔积液患者，差异具有统计学意义，表明其可用于良、恶性胸腔积液鉴别诊断。Liao等^[26]^用ELISA测定97例患者不同病因导致的胸腔积液中s-CAIX含量，结果同样证明：恶性胸腔积液组s-CAIX含量明显高于良性胸腔积液组。依据s-CAIX浓度绘制ROC曲线，曲线下面积为0.761(95%CI: 0.622-0.901, *P*=0.002)。当阈值109.135 pg/dL，敏感度和特异度分别为92.3%及58.3%，恶性胸腔积液组仅1例患者胸腔积液中s-CAIX含量低于该阈值(87.09 pg/mL)。上述结果提示，胸腔积液中s-CAIX含量检测对鉴别良、恶性胸腔积液具有较好的诊断价值。

胸腔积液沉淀物取材方便，患者痛苦小，易接受，且可反复多次取材送检，近些年被广泛用于恶性胸腔积液的诊断。本研究收集21例肺癌恶性胸腔积液标本，其胸腔积液沉淀物CAIX表达阳性率为66.67%，且特异性为100%，而结核组CAIX表达均阴性。Liao等^[26]^研究结果也证明，良性胸腔积液组沉淀物均不表达CAIX，而63.6%(21/33)的恶性胸腔积液沉淀物明显表达，与本研究结果相近。Li等^[27]^用RT-PCR技术测定71例胸腔积液中*CAIX*的基因表达，结果显示，89.8%(53/59)的恶性胸腔积液中有*CAIX*基因表达，而在良性对照组中仅有8.1%(1/12)表达。Liao等^[28]^通过免疫细胞学染色，对150例胸腔积液细胞涂片进行研究，结果表明，良性胸腔积液组均不表达CAXI和GLUT1，而恶性胸腔积液组CAIX和GLUT1的阳性率分别为63.8%、74.5%，且特异性为100%。可疑恶性胸腔积液组和不典型胸腔积液组中细胞学为阴性，但CAIX或(和)GLUT1阳性的12例(其中CAIX阳性11例，GLUT1阳性7例)，最终均被证明为恶性胸腔积液。综合文献报道及本研究可以认为，胸腔积液沉淀物CIAX染色对良、恶性胸腔积液具有较高的鉴别诊断价值，且对于怀疑为恶性胸腔积液但细胞学为阴性者，使用胸腔积液沉淀物检测CAIX的表达可明显提高诊断率^[29]^。

综上所述，胸腔积液中s-CAIX的测定及胸腔积液沉淀物中CAIX的免疫组化染色，对恶性胸腔积液的诊断有较高的临床价值。
